# Physiological and Epigenetic Features of Yoyo Dieting and Weight Control

**DOI:** 10.3389/fgene.2019.01015

**Published:** 2019-12-11

**Authors:** Raian E. Contreras, Sonja C. Schriever, Paul T. Pfluger

**Affiliations:** ^1^Research Unit Neurobiology of Diabetes, Helmholtz Zentrum München, Neuherberg, Germany; ^2^Institute for Diabetes and Obesity, Helmholtz Zentrum München, Neuherberg, Germany; ^3^German Centre for Diabetes Research (DZD), Neuherberg, Germany; ^4^Neurobiology of Diabetes, TUM School of Medicine, Technische Universität München, Munich, Germany

**Keywords:** weight loss, Yoyo dieting, CNS, epigenetic mechanisms, obesogenic memory

## Abstract

Obesity and being overweight have become a worldwide epidemic affecting more than 1.9 billion adults and 340 million children. Efforts to curb this global health burden by developing effective long-term non-surgical weight loss interventions continue to fail due to weight regain after weight loss. Weight cycling, often referred to as Yoyo dieting, is driven by physiological counter-regulatory mechanisms that aim at preserving energy, *i.e.* decreased energy expenditure, increased energy intake, and impaired brain-periphery communication. Models based on genetically determined set points explained some of the weight control mechanisms, but exact molecular underpinnings remained elusive. Today, gene–environment interactions begin to emerge as likely drivers for the obesogenic memory effect associated with weight cycling. Here, epigenetic mechanisms, including histone modifications and DNA methylation, appear as likely factors that underpin long-lasting deleterious adaptations or an imprinted obesogenic memory to prevent weight loss maintenance. The first part summarizes our current knowledge on the physiology of weight cycling by discussing human and murine studies on the Yoyo-dieting phenomenon and physiological adaptations associated with weight loss and weight re-gain. The second part provides an overview on known associations between obesity and epigenetic modifications. We further interrogate the roles of epigenetic mechanisms in the CNS control of cognitive functions as well as reward and addictive behaviors, and subsequently discuss whether such mechanisms play a role in weight control. The final two parts describe major opportunities and challenges associated with studying epigenetic mechanisms in the CNS with its highly heterogenous cell populations, and provide a summary of recent technological advances that will help to delineate whether an obese memory is based upon epigenetic mechanisms.

## The Physiology of Weight Cycling

Obesity and its deadly comorbidities such as type-2 diabetes (T2D), cardiovascular diseases or some forms of cancer are health problems of the first order (World Health Organization, 2018). In 2016, more than 1.9 billion adults and 340 million children were classified as overweight or obese. However, efforts to curb the ever-increasing obesity pandemic have largely failed.

Non-surgical therapies against obesity are ineffective for a large proportion of obese individuals. Studies in overweight and obese individuals undergoing weight loss reported that only 20% of the participants were able to maintain the weight loss long term (McGuire et al., 1999; Wing and Phelan, 2005). Thus, consistent with reports that 2–6% body weight regain can already reverse the benefits of losing ≥10% body weight (Kroeger et al., 2014), novel strategies against the regain in weight are of utmost need.

### Defining Successful Weight Loss

A successful weight loss maintainer is defined as an individual who voluntarily decreases ≥10% of the initial body weight and keeps the lost weight for ≥1 year (Wing and Hill, 2001). Most individuals fail in maintaining their weight loss due to weight cycling, often referred to as Yoyo dieting. Weight regain often starts within the first year, and the pre-intervention weight is reached or even surpassed in the subsequent 2 to 5 years (Anderson et al., 2001; Weiss et al., 2007). Lean individuals that were voluntarily overfed with 50% additional calories for 3 days showed decreased pre-meal hunger and increased post-meal satiety (Cornier et al., 2004). In obese individuals that underwent weight loss, overfeeding did not diminish hunger or increase satiety. This absence of compensatory changes in hunger and satiety upon overfeeding likely contributes to an increased propensity for weight regain in obese individuals that undergo weight loss (Cornier et al., 2004). Overall, only 11% of the individuals with early-onset weight re-gain can achieve a subsequent body weight loss within that first year (Wing and Phelan, 2005).

Limiting or preferentially avoiding weight cycling in the first year after weight loss appears to be crucially important for a sustainable long-term weight maintenance, as evidenced by a study that reported a 50% decrease in the risk for subsequent weight regain in individuals that managed to maintain their successful weight loss for 2 years (Wing and Phelan, 2005). Most therapeutic strategies against this first-year Yoyo-dieting-effect are built upon nutritional interventions, *i.e.* calorie and/or fat restriction, ketogenic diets or intermittent fasting. These strategies can be highly efficacious, as evidenced by the National Weight Control Registry (NWCR), an ongoing longitudinal study of more than 4,000 successful weight loss maintainers (≥13.6 kg (30 lb) for ≥1 y) (Wing and Phelan, 2005; Bond et al., 2009). Strict adherence to weight loss maintenance strategies appears to be key for 89% of these successful weight loss maintainers, which includes both high levels of physical activity and consuming a low calorie, low fat diet.

### Physiological Adaptations to Weight Loss

Body weight maintenance requires a dynamically adjusted homeostasis of energy intake and energy expenditure. A chronically negative energy balance would lead to the depletion of energy stores, a chronically positive energy balance to an undesired accumulation of energy surplus (Maclean et al., 2011). Unfortunately, in our modern Westernized societies a chronically positive energy balance is the norm for many. Stressful and sedentary lifestyles are combined with an overconsumption of highly palatable and energy-dense food enriched in saturated fats and processed sugars. The excess of energy intake leads to the development of overweight and ultimately to obesity (Melby et al., 2017). Becoming obese does not occur overnight. It takes a considerable amount of time under constant obesogenic pressure to develop adiposity. This length in time also allows an individual's biology to adapt to the new state of obesity (Corbett et al., 1986). This adaptive process defines a state in which energy expenditure and high energy intake are balanced to defend the newly gained weight and adiposity (Corbett et al., 1986; Kirchner et al., 2012).

To lose weight, obese individuals often undergo severe caloric restriction, *i.e.* they reduce their overall energy intake to create a negative energy balance (Rosenbaum et al., 2010). In consequence, the body readily adapts by a rapid decrease in the total daily energy expenditure (TDEE) to preserve energy and restore homeostasis (Rosenbaum et al., 2008). This decrease in TDEE can nevertheless be disproportionate to the decrease in energy intake, as evidenced by a report that showed 25% lower TDEE in weight-reduced compared to never-obese individuals (Leibel et al., 1995). By the end of a weight loss period, all three main components of TDEE are reduced, *i.e.* the thermic effect of food required for the digestion and absorption of ingested calories (Maclean et al., 2011), activity-induced energy expenditure including non-exercise activity thermogenesis (NEAT) and exercise energy expenditure (EEE) (Goldsmith et al., 2010; Hames et al., 2016), and the resting metabolic rate (RMR) (Melby et al., 1990; Astrup et al., 1999; Doucet et al., 2001). The reduction in TDEE after profound weight loss can last for several years (Camps et al., 2013) and impairs the long-term maintenance of weight loss in both mice and men (Hill et al., 1987; Froidevaux et al., 1993; Maffei et al., 1995; Doucet et al., 2001; MacLean et al., 2004). For instance, participants of the TV show "The Biggest Loser" showed a persistent decrease in their RMR even 6 years after the weight loss, which likely contributed to the regain in body weight in all but one of the 14 subjects (Fothergill et al., 2016).

The arguably most important factor that determines weight maintenance vs. weight re-gain after weight loss is food intake. Our ingestive behavior is built upon parallel and complementary mechanisms that integrate peripheral signals from circulating hormonal factors for hunger or fullness with homeostatic feeding circuitry in the hypothalamus and brain stem and hedonic processes that are partially beyond our cognitive control (Waterson and Horvath, 2015). Weight loss by calorie restriction is associated with increased hunger and a strongly increased reward value of food (Rosenbaum et al., 2010; Burger and Stice, 2011; Blundell et al., 2012; Caudwell et al., 2013). Notably, the sensation of increased hunger appears to persist beyond the phase of rapid weight loss; previously obese mice that had been subjected to rapid weight loss by calorie restriction showed hyperphagia when re-fed *ad-libitum* with chow fed diet, leading to accelerated weight re-gain even when compared to never-obese mice subjected to a HFD (Kirchner et al., 2012). In contrast, when diet-induced obese mice were subjected to slow weight loss induced by *ad libitum* low calorie diet feeding, hyperphagia was absent and the mice maintained their reduced body weight (Fischer et al., 2018).

### Hormonal Factors Guide Our Compensatory Response to Weight Loss

Mechanistically, weight loss was shown to induce a profound deregulation of circulating nutrients such as glucose or free fatty acids which can act as signaling moieties in CNS centers governing energy and glucose homeostasis (Lam et al., 2005; He et al., 2006). Similarly, weight loss and a negative energy balance altered the secretion of circulating hormones such as the orexigenic ghrelin or the anorexigenic leptin, insulin, GLP-1, CCK and PYY (Maffei et al., 1995; Näslund et al., 2000; Crujeiras et al., 2010; Rosenbaum et al., 2010; Maclean et al., 2011; Melby et al., 2017). These endocrine adaptations to a chronically negative energy balance are believed to drive hyperphagia and the frequency and/or the size of meals in both humans and rodents (Kirchner et al., 2012; Karatsoreos et al., 2013; Catenacci et al., 2014). Such altered levels of circulating hormones that facilitate weight regain appear to persist for at least 1 year (Sumithran et al., 2011; Camps et al., 2013). Accordingly, numerous attempts have tried to utilize hormone replacement interventions to combat weight-loss induced physiological adaptations, *i.e.* the increase in hunger and decrease in energy expenditure (Kelesidis et al., 2010; Kissileff et al., 2012; Trujillo et al., 2015; Vodnik et al., 2016). For instance, leptin replacement was considered as putative antidote against weight regain based on a clinical study that showed promising benefits of leptin replacement in weight loss patients, *i.e.* increases in sympathetic nerve system activity and a restoration of skeletal muscle work efficiency (Rosenbaum et al., 2005). Similarly, mice undergoing leptin replacement therapy displayed an increased expression of the anorexigenic neuropeptide proopiomelanocortin and a normalized energy homeostasis and weight maintenance (Chhabra et al., 2016). However, these reports stand in contrast to a chronic leptin infusion study in mice which suggested that the weight maintenance is independent from leptin (Ravussin et al., 2014).

In summary, weight loss is associated with adaptive physiological processes that evolved to defend body weight from weight loss, *i.e.* increased hunger and food reward behavior and decreased energy expenditure. The signals that drive these changes may be hormonal, but their exact nature remains elusive. How weight loss is achieved appears to be of crucial importance. If the negative energy balance stems from severe caloric restriction, strong counter-regulatory stimuli come to play. A slow weight loss, *e.g.* based on switching from an obesogenic to a healthier diet, seems to evade some of the adaptive responses to fully reverse obesity. However, at current this paradigm of fast vs. slow weight loss still lacks solid experimental proof and a theoretical foundation.

### The Set-Point Theory Postulates an Obesogenic Memory Against Weight Loss

The long persistence of metabolic adaptations in response to rapid weight loss in obese individuals raises a critical question: is there an acquired obesogenic memory that drives weight regain in order to maintain a previous status quo? Many hypotheses have been postulated regarding 'body weight points' and acting compensatory mechanisms. One of the most debated ones, the set-point or lipostatic theory, was first introduced by Kennedy in the early 50s (Kennedy and Sterling, 1953). A fat derived signal, conveying the status of fat-depots, is sensed by the brain where it is compared to a target level, in order to trigger compensatory mechanisms upon any disturbance. This negative feedback loop was strongly supported by the discovery of leptin (Zhang et al., 1994), genetic mutations in the leptin gene and the MC4R family (Farooqi et al., 1999; Farooqi and O'Rahilly 2008) and the description of leptin resistance in diet-induced obese mice and obese humans (Maffei et al., 1995; Schwartz et al., 1996; Halaas et al., 1997; Friedman 2016). However, in this past decade several studies questioned the existence of leptin resistance (Ottaway et al., 2015; Harrison et al., 2018; Pan and Myers Jr, 2018). Moreover, leptin infusion studies in mice revealed that hyperleptinemia is unlikely to define an obese body weight set point (Ravussin et al., 2014).

The set-point theory remains to be controversially discussed, with both reports corroborating a set point in human and animal studies, and multiple reports that find no experimental evidence for a set point (Halaas et al., 1997; Carhuatanta et al., 2011; Ravussin et al., 2014; Chhabra et al., 2016; Jansson et al., 2018). Models describing single factors fail to fully explain the observed weight cycling phenomenon. As an alternative concept, the dual intervention point theory uses two independent upper and lower weight levels as boundaries for active regulation. A lower weight boundary is indispensable to avoid starvation in times of low energy availability, and compensatory mechanisms that counteract starvation and weight loss are evolutionary conserved and privileged. An upper weight level was delimited by the risk of predation that required a certain leanness and fitness. However, around 2 million years ago early humans evolved enough to partially release the risk for predation (Speakman, 2018), which liberated the upper weight level and mechanisms that prevented from excessive weight and fat accumulation. The phenomenon, also described as genetic drift, has been suggested as a major driver for the obesity epidemic (Speakman, 2007).

### Resetting the Obesogenic Memory by Bariatric Surgery

At current, there is only one efficient therapeutic strategy against obesity: bariatric surgery. In essence, bariatric surgery is the reorganization of the gastrointestinal track that not only reverses severe obesity, but also helps patients with moderate degrees of obesity and difficult-to-control metabolic disorders (Lutz and Bueter, 2014). Two of the most commonly applied bariatric surgeries, Roux-en-Y gastric bypass (RYBG) and vertical sleeve gastrectomy (VSG), successfully achieve approximately 25% weight loss within the first year. Contrary to the persistently decreased RMR observed in weight loss patients undergoing calorie restriction, RYGB individuals showed a temporarily reduced RMR which resolved to normal after 1 year (Knuth et al., 2014; Fothergill et al., 2016). A comparison between successful weight losers *via* surgical or non-surgical interventions confirmed that weight loss maintenance is achievable to the same degree in both cases, but surgical-patients require less physical activity and dietary restraint (Bond et al., 2009). Similarly, weight regain mechanisms such as increased hunger and decreased energy expenditure were absent in RYGB rodent models (Bueter et al., 2010; Hao et al., 2013).

Mechanisms mediating the beneficial effects of bariatric surgery are largely elusive, but first evidence points to an improved communication between peripheral organs and the CNS, possibly mediated *via* hormones, biles acids and/or neuronal pathways. For instance, mice with a deficiency for farnesoid X receptor (FXR), a transcription factor activated by bile acids, were reported to be unresponsive to bariatric surgery (Ryan et al., 2014). FXR KO mice showed full post-surgical weight regain 11 weeks after VSG surgery, while wildtype littermates showed sustained weight loss (Ryan et al., 2014). In both humans and rodents, bariatric surgery alters the composition and concentration of bile acids (Patti et al., 2009; Kohli et al., 2013; Myronovych et al., 2014). Accordingly, it appears possible that benefits of bariatric surgery such as improved glucose disposal and lipid homeostasis are in part mediated by bile acids and FXR activation, potentially driven *via* an increase in post-prandial FGF19 secretion (Schaap, 2012; Wu et al., 2013). Bariatric surgery is furthermore associated with profound alterations in the post-prandial release of anorexigenic (*e.g.* GLP-1 and PYY) and orexigenic (ghrelin) gastrointestinal hormones. These changes in the secretion are often discussed as likely mechanisms for metabolic benefits of bariatric surgery, as evidenced by the lack of effects of bariatric surgery in mice deficient for PYY (le Roux et al., 2006). However, studies in GLP1 and ghrelin wild-type and knockout mice showed comparable weight loss after bariatric surgery, respectively (Chambers et al., 2013; Wilson-Pérez et al., 2013; Mokadem et al., 2014; Ye et al., 2014). Leptin replacement therapy has recently been shown to improve the metabolic response of leptin-deficient (Lep^ob^) mice to RYGB surgery (Hao et al., 2015). In humans, the decrease in circulating leptin levels is smaller after RYGB (∼50%) compared to lifestyle interventions (∼100%) (Knuth et al., 2014). Thus, an improvement in leptin sensitivity seems like a tempting hypothesis, but studies in VSG-operated rats showed no difference in leptin sensitivity when compared to pair-fed control rats (Stefater et al., 2010). Notwithstanding, the melanocortin system and its melanocortin 4 receptor (MC4r) are required for the benefits of RYGB in energy expenditure, body weight and glucose metabolism in mice (Hatoum et al., 2012; Mokadem et al., 2014). Similarly, human carriers of a specific MC4R gene variant [MC4R(I531L)] showed an improved outcome to bariatric surgery with a better metabolic status (Zechner et al., 2013). Overall, our understanding on whether peripheral hormones mediate the effects of bariatric surgery, either as single entities or in combination, is still incomplete. Additional evidence points towards a direct interaction with CNS melanocortin signaling, but details remain to be clarified.

## The Epigenetics of Obesity and Weight Cycling

**Figure 1:**
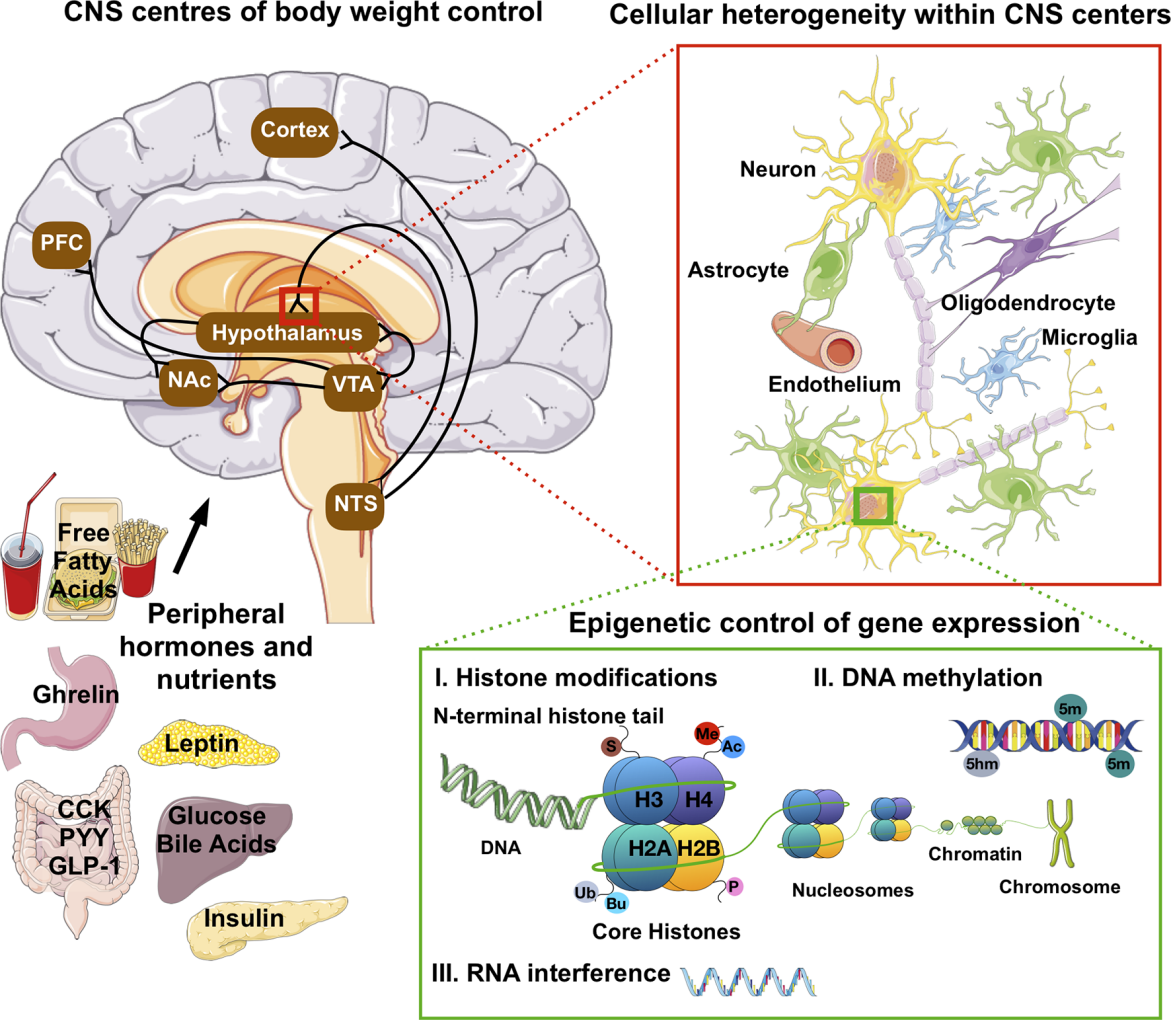
Anatomical, cellular and molecular heterogeneity in the CNS control of food intake and energy homeostasis. Hormonal and nutrient cues from peripheral tissues signal the state of our dietary and energetic requirements to brain regions that govern our homeostatic and hedonic control of food intake and body weight. These neuronal circuits are deeply interconnected, and embedded within a network of vascular cells and highly diverse glial cell populations ranging from astrocytes to microglia and oligodendrocytes. The lineages and identities of these highly diverse neuronal and glial subpopulations are in part driven by variations in the epigenome, *i.e.* epigenetic changes such as DNA methylation, histone post-translation modifications (Ac, acetylation; Me, methylation-Ub, ubiquitination; P, propionylation; Bu, butyrylation and S, sumoylation) and RNA-mediated post-translational interference. In fully developed, post-mitotic neurons, epigenetic mechanisms were further shown to influence reward behaviors and cognitive functions. PFC, pre-frontal cortex; NAc, nucleus accumbens; VTA, ventral tegmental area; NTS, nucleus of the solitary tract; CCK, cholecystokinin; PYY, peptide YY; GLP-1, glucagon-like peptide 1.

Epigenetic modifications are reversible alterations in gene transcription without alterations in the underlying genomic DNA sequence. Current epigenetic modifications include 1) DNA methylation, *i.e.* the covalent attachment of a methyl group to the 5 position of a cytosine (5 mC) or its conversion into 5-hydromethylcytosine (5hmC) in CpG and non-CpG islands; 2) histone posttranslational modifications (HPTM), *i.e.* the addition of a variety of molecules to the protruding tails of the core histones and 3) RNA interference mediated post-transcriptional modifications (**Figure 1**). Together, epigenetic mechanisms modulate transcriptional programs by promoting chromatin remodeling around genomic regions that can enhance or inhibit gene transcription (Bird, 1999; Egger et al., 2004; Day and Sweatt, 2011; Smallwood and Kelsey, 2012).

### Obesity Is Associated With Distinct Epigenome Signatures in Peripheral Tissues

Epigenetic modification can be self-perpetuating and heritable, as shown by multiple transgenerational studies on inheritable traits (Egger et al., 2004; Seki et al., 2012; Dalgaard et al., 2016). Such evidence supporting the role of epigenetics in developmental programming of obesity has for instance been demonstrated in whole blood of patients that were born to women exposed periconceptually to severe undernutrition in the Dutch Hunger during World War II (Heijmans et al., 2008). At the age of 58 years, these patients showed reduced methylation of the imprinted gene *IGF2* (Insulin-like growth factor 2) and a higher risk of obesity or glucose intolerance (Ravelli et al., 1998; Stein et al., 2007). Animal studies confirmed the impact of under- or overnutrition before or during gestation on the epigenome and the phenotype of the offspring (Zhang et al., 2010; Ainge et al., 2011; Nicholas et al., 2013).

Recent studies further demonstrate the importance of reversible non-heritable epigenetic modifications in adulthood and in post-mitotic and fully differentiated cells (Renthal et al., 2007; Day and Sweatt, 2011; Lester et al., 2011; Vucetic et al., 2011; Vucetic et al., 2012). Associations between obesity and epigenetic changes, mostly in DNA methylation, have for instance been reported for blood cells such as peripheral blood leukocytes (PBLs) as well as muscle, adipose tissue, placenta and colon. Candidate-based gene methylation studies have identified multiple differentially methylated genes in blood and peripheral tissues of obese compared to lean individuals. Reduced methylation of tumor necrosis factor (*TNFa*) (Hermsdorff et al., 2013) and the adipocyte-derived hormone leptin (*LEP*) (Obermann-Borst et al., 2013) were found in blood of obese humans, whereas methylation of circadian rhythm protein CLOCK : BMAL1 (Circadian Locomotor Output Cycles Kaput and aryl hydrocarbon receptor nuclear translocator-like) was increased by adiposity in PBLs (Milagro et al., 2012). In muscle of obese humans, methylation of pyruvate dehydrogenase kinase 4 (*PDK4*) was decreased but methylation of PPARγ coactivator 1 alpha (*PGC1α*) was increased compared to lean individuals (Barres et al., 2013). Recently, three lines of evidence—epigenetic dysregulation following high fat diet in mouse, epigenetic directional consistency in humans, and some evidence for clinical risk of T2D—were combined to identify epigenetic mechanisms in adipose tissue that most likely have a functional implication in the pathogenesis of obesity-driven T2D (Multhaup et al., 2015). The identified candidate genes *MRTFA* (myocardin related transcription factor A), *PLEKHO1* (pleckstrin homology domain containing O1) and *TNFAIP8L2* (TNF alpha induced protein 8 like 2) were all hypomethylated in HFD-fed mice and obese humans and had increased gene expression. Moreover, overexpression of these genes in cell culture adipocytes decreased uptake of glucose in response to insulin which is consistent with an increased insulin resistance commonly observed in obesity and diabetes (Multhaup et al., 2015). However, most of these studies are cross-sectional and epigenetic changes were recorded at the same time-point as the obese phenotype. Accordingly, to determine whether epigenetic modifications such as DNA methylation marks are a cause or consequence of obesity remains a challenge in the field (van Dijk et al., 2015).

The study of epigenetic mechanisms in weight control is further complicated by the recent finding that DNA methylation profiles are instable in adulthood and affected by diet, exercise and weight loss interventions (Milagro et al., 2011; Rönn et al., 2013; Multhaup et al., 2015). DNA methylation profiles of blood cells from obese patients, successful weight loss maintainers and never-obese individuals showed marked differences in the genes *BDNF* (brain derived neurotrophic factor), *RIR1* (ribonucleotide reductase catalytic subunit M1), and *TUBA3C* (tubulin alpha 3c) (Huang et al., 2015). Interestingly, after weight loss surgery muscle DNA methylation profiles of formerly obese individuals became more similar to those of the lean controls (Barres et al., 2013) indicating that some methylation marks are rather a consequence of than a predisposition for obesity. It was further shown that adipose tissue DNA methylation profiles can not only be modulated by exercise (Rönn et al., 2013) but can also be used to predict the response to weight loss interventions in humans (Bouchard et al., 2010; Cordero et al., 2011). In the liver of diet-induced obese (DIO) mice, treatment with metformin, a widely used drug against T2D, reversed the increased histone methylation (H3K36me2) (Nie et al., 2017) indicating that pharmacological interventions can also affect epigenetic mechanisms at the level of the chromatin.

Overall, growing evidence suggests that epigenetic changes are mechanistically linked with the regulation of body weight. However, causality for this phenomenon remains to be provided. Moreover, whether any of the recently identified genes contribute to the heritability of obesity has not been established. Here, the recent identification of reduced expression of an imprinted gene network including the genes neuronatin (*Nnat*), paternally expressed 3 (*Peg3*), cyclin-dependent kinase inhibitor 1C (*Cdkn1c*) and pleiomorphic adenoma gene-like 1 (*Plagl1*) in the obese population provided genetic evidence that mechanisms exist in mammals to canalize developmental and phenotypic outcomes along discrete trajectories (Dalgaard et al., 2016).

### Epigenetic Mechanisms Regulate the CNS Control of Behavior

Post-mitotic epigenetic modification are an integral process for neurons during memory formation and learning. Memory consolidation during contextual fear learning increased global di-methylation of histone 3 lysine 9 (H3K9me2) in the CA1 region of the hippocampus and the lateral entorhinal cortex, with marked transcriptional regulation on genes like *Bdnf*, *Fos*, *Dnmt3a* and *Zif268* at both the level of the promoter and mRNA expression(Gupta-Agarwal et al., 2012). Modifications of the H3K9me2 histone lysine methyltransferase G9a (H/KMTs-G9a) or demethylase LSD1 (H/KDMs-LSD1) in the lateral amygdala (LA) were shown to impair or enhance fear memory consolidation, respectively (Gupta-Agarwal et al., 2014). Similarly, DNA methylation contributed to maintain long-term (remote) memories (Miller et al., 2010). Persistent DNA hypomethylation in CpG islands at the *Bdnf* locus was functionally linked with the specific upregulation of exons I and IV in the adult hippocampus and a consolidation of fear memory (Lubin et al., 2008; Mizuno et al., 2012).

Next to the effects on memory formation and learning, epigenetic modifications in neurons have been associated with cognitive impairment in response to psychostimulant and drug abuse, and with a direct impact on reward behavior. Upon chronic cocaine exposure, histone deacetylase 5 (HDAC5) in the nucleus accumbens (NAc) was shown to mitigate H3-hyperacetylation at gene promoters that ultimately lead to gene activation (*i.e.* NK1R upregulation) and the promotion of reward behaviors within a conditioned place preference paradigm (Renthal et al., 2007). Methamphetamine intake promoted hydroxylation and hypomethylation in CpG-rich regions in the promoter and intragenic sequences of the *Crh/Crf* and *Avf* genes in the NAc (Jayanthi et al., 2018). The increased expression of both neuropeptide genes is associated with prolonged neuroendocrine responses like hypersecretion of plasma adrenocorticotropine hormone (ACTH) and cortisone after acute amphetamine injections (Bisagno and Cadet, 2014). In the medial prefrontal cortex (mPFC), methamphetamine increased the expression of *Drd1* and *Grin1* by augmenting H4ac enrichment at their promoter regions but decreased expression of *Hcrtr2* by reducing H3ac enrichment at its promoter region (González et al., 2018). Overall, these studies highlight the role of post-mitotic epigenetics in conveying long-term adaptations to the environment.

### Epigenetic Mechanisms in the CNS Control of Food Intake and Body Weight

Given the involvement of epigenetic mechanisms in cognition and reward behaviors, it seems plausible that epigenetic mechanisms may also be linked to the CNS hedonic control of food intake and food reward behaviors, and in general to the development of obesity in both the CNS and peripheral tissues. However, few studies have addressed whether post-mitotic epigenetic modifications upon chronic exposure to an obesogenic environment exist within the CNS, and whether they play a functional role in the etiology of metabolic dysfunction. For peripheral tissues, such distinct epigenome signatures have been reported, as outlined above. For the CNS, we know that multiple epigenetic regulators are expressed in brain regions involved in feeding behaviors and energy homeostasis such as the hypothalamus, the midbrain or the brain stem. However, whether they are functionally involved in the CNS regulation of energy and glucose metabolism largely remains to be determined.

Obesity-induced changes in the epigenetic marks of the CNS were investigated in a study by Vuceti et al., (2011) who correlated a decrease in µ-opioid receptor gene expression in reward circuitry of the ventral tegmental area (VTA), NAc and PFC with epigenetic repressive marks such as increased DNA methylation in the promoter and binding sequence of methyl CpG binding protein 2 (MeCP2), increased H3K9 methylation and decreased H3 acetylation in obese mice compared to lean controls. Similarly, DIO mice showed a reduced DNA methylation in the tyrosine hydroxylase (TH) and dopamine transporter (DAT) in the hypothalamus which was correlated with increased expression of both mRNAs (Vucetic et al., 2012). The exact opposite pattern on DNA methylation and mRNA expression of DAT and TH was evidenced in the VTA. Moreover, dopamine receptors D1 and D2 were also decreased in the striatum which complemented the dopaminergic dysfunction in obesity (Alsiö et al., 2010).

Overall, there is emerging evidence that environmental changes in response to an obesogenic environment can induce epigenetic modifications in multiple tissues and cell types. Several studies suggest an impact of epigenetic modifications on reward circuitry involved in our hedonic control of feeding. Nonetheless, at current there is no evidence on whether and how epigenetic mechanisms can converge into a compulsive phenotype imprinted in an obesogenic environment.

## Opportunities and Challenges in Studying Cns Epigenetics

The relative lack of studies on epigenetic mechanisms in the CNS control of food intake and metabolism is based on a number of factors such as the complexity of the brain with its high cell heterogeneity. Nonetheless, at current our knowledge on the epigenome and transcriptome of neuronal as well as glial CNS subpopulations is propelled by technological advances in next generation sequencing and computational analysis, as outlined below.

### Cellular Heterogeneity in CNS Control Centers of Body Weight Homeostasis

Numerous neuronal subpopulations are part of a complex and incompletely understood network that orchestrates our metabolic homeostasis (**Figure 1**). Next to neurons, there is a multitude of glial cell types, ranging from oligodendrocyte species to astrocyte and microglial subpopulations. These are embedded within a dense network of blood vessels with endothelial cells and pericytes, and form a functional blood brain barrier interface that plays a vital role in nutrient (*e.g.* glucose) and endocrine (*e.g.* insulin and leptin) sensing and the CNS control of feeding behavior and energy homeostasis as well as brain glucose uptake (Kim et al., 2014; García-Cáceres et al., 2016).

All these cell types contain the same copy of the genome, but they greatly differ in the spatial and temporal expression of defined portions of the genome that define and drive the lineage of the respective cellular sub-population. Isolating RNA or DNA from a specific CNS cell type nonetheless remains a major experimental challenge due to the fragility and high interconnection. Accordingly, most of the research on adult CNS has been limited to the analysis of epigenetic variations in bulk brain tissue. Pooling multiple cell types together likely masks cell type-specific variations and requires complex bioinformatical deconvolution algorithms. Gene co-expression modules, *i.e.* co-expressed genes of a cell type that are highly correlated to the proportion of the cell type in a sample and deduced from a large number of independent datasets, permit to determine core transcriptional features of specific cell-types (Kelley et al., 2018). Additional algorithms such as MetaVIPER (Ding et al., 2018) and machine learning tools to leverage large data sets will help to overcome the challenges associated with cellular heterogeneity (Tritschler et al., 2017; Eraslan et al., 2019; Fischer et al., 2019). However, whether they are universally applicable to specific brain-region and cell-type analyses remains underexplored.

### Novel Tools to Isolate and Study Defined CNS Subpopulations

Several approaches can be used to dissociate and isolate specific CNS populations for downstream epigenome analyses. Fluorescence activated cell sorting (FACS) and magnetic activated cell sorting (MACS) in combination with specific antibody panels are now used since more than 30 years to isolate the three main brain cell types, *i.e.* neurons, astrocytes and microglia (Paden et al., 1986; Martin et al., 2017). However, working with fresh tissue requires enzymatic dissociation and a time-consuming sorting, which can result in low cell recovery and artefactual stress responses and gene expression patterns. Embryonal and neo-natal brains appear to be more suitable for FACS/MACS of whole cells, but the approach has also been used in adult rodents (García-Cáceres et al., 2016; Paeger et al., 2017). For instance, in post-natal forebrains, transcriptome profiling of FACS-sorted neurons, astrocytes and oligodendrocytes revealed common metabolic pathways, but also a high variety and several highly dissimilar sub-clusters within the glial cell class (Cahoy et al., 2008). Differences in transcriptome profiles are likely reflected by differences in gene regulation, as highlighted by specific cell-type DNA methylation profiling in hypothalamic post-natal neurons versus glia (Li et al., 2014).

The dissociation and isolation of nuclei from frozen or fixed tissue has shown the potential to overcome the challenges associated with isolating intact cells from fresh tissue (Jiang et al., 2008; Krishnaswami et al., 2016). Nuclei are stable and largely devoid of cytosolic factors that interfere with epigenomic processes. Moreover, their protein-coding transcript content is highly reminiscent of the content in whole cells (Barthelson et al., 2007; Solnestam et al., 2012; Grindberg et al., 2013). Nuclei further express specific membrane-bound antigens such as NeuN or OLIG2 which mark them as neuronal or oligodendrocyte nuclei, respectively (Xu et al., 2018b). By applying specific antibodies and FACS or MACS, these nuclear subpopulations can be sorted and characterized from adult brain tissues. This approach can further be advanced by using genetically engineered mice with a nuclear membrane-bound fusion protein (Sun1-sfGFP-Myc) that is expressed under the control of Cre recombinase (Deal and Henikoff, 2011; Mo et al., 2015). The fusion protein on the nuclear membrane is only expressed in Cre-positive CNS sub-populations, and nuclei with the fusion protein can be isolated from brain areas by applying the protocol for the isolation of nuclei tagged in specific cell types (INTACT). This INTACT protocol allows to isolate both DNA and RNA from the nuclei of rare CNS sub-populations for a full transcriptome and epigenome profiling (Mo et al., 2015). An alternative approach, the translating ribosome affinity purification (TRAP) technology, is directed towards the selective enrichment of RNA bound to ribosomes of genetically tagged CNS subpopulations (Doyle et al., 2008). The TRAP method is based on a genetic mouse model with an eGFP-L10a ribosomal fusion protein under the control of Cre recombinase, and is restricted to the analysis of the translatome within Cre-positive cell populations (Heiman et al., 2008).

Next to recent advances in methods and genetic tools to isolate CNS sub-populations, downstream next generation sequencing (NGS) applications have undergone major technological improvements to also allow the transcriptome and epigenome profiling of low input material. Regular high-throughput NGS protocols continue to require millions of cells, which goes beyond the typical yield when working with small brain regions and rare cellular CNS sub-populations. However, in the last years single cell RNAseq became a new standard in the field and is now widely used to interrogate cellular identities and lineages of whole organisms (Plass et al., 2018), whole peripheral organs (Tritschler et al., 2017; Angelidis et al., 2019) or small brain regions (Zeisel et al., 2015; Lam et al., 2017; Moffitt et al., 2018). Multiple efforts are further underway to enable single cell epigenome profiling (Kelsey et al., 2017; Lake et al., 2018; Wen and Tang, 2018)) but they are not yet reaching the informational depth of scRNAseq. Nonetheless, new NGS methods such as single cell ATACseq (Jia et al., 2018), low-input whole-genome bisulfite sequencing (Peat and Smallwood, 2018), Cut&Run (Skene and Henikoff, 2017) and (HT-)ChIPmentation (Schmidl et al., 2015; Gustafsson et al., 2019) can bridge the gap between single cell resolution and bulk tissue profiling, and enable to interrogate the transcriptome and epigenome of rare CNS sub-population in unprecedented detail.

## Summary and Outlook

Losing weight and remaining lean is a very hard task for most of the overweight and obese individuals (Wing and Phelan, 2005). The principal issue is not losing weight *per se*, but rather the fight against physiological adaptions directed against keeping the lost weight (Maclean et al., 2011; Dulloo and Montani, 2015). Bariatric surgery is currently the only option to sustainably lose weight, but its mechanisms are largely unclear (Knuth et al., 2014). The CNS with homeostatic control centers within the hypothalamus and hindbrain appears to be key for the maintenance of body weight. Homeostatic brain centers sense and integrate a plethora of peripheral and environmental messages to adequately regulate food intake and energy homeostasis (Waterson and Horvath, 2015). Of similar importance are hedonic control centers and reward circuitry within the limbic system, which orchestrate behavioral and biological responses such as food seeking, food reward and food perception behaviors, memory formation and cognition. These brain regions are susceptible to epigenetic reprogramming (Alsiö et al., 2010; Miller et al., 2010; Gupta-Agarwal et al., 2014; Jayanthi et al., 2018), and are likely targets for epigenetic modifications induced by obesity. Future studies should unravel whether an epigenetic memory for obesity exists within these CNS centers, and whether it is functionally linked to the regulation of body weight or weight re-gain after weight loss. The large heterogeneity of cell types in the CNS makes this a particularly challenging endeavor. Even neuronal subpopulations within a small region can show high diversity (Quiñones et al., 2018; Xu et al., 2018a). Moreover, neurons with their complex morphology based on long axonal connections and dendritic arborization show a high degree of interconnection and interspersion with other neuronal subpopulations and multiple glial cell types. Isolating and sorting individual CNS cell types can be achieved on a single cell (SC) and single nucleus (SN) level by FACS or immunopanning (IP). Genetic models that label individual cell types with a reporter can greatly improve this initial isolation and sorting step and subsequent epigenome profiling. Technological advances such as single cell RNAseq, ChIPmentation or Cut&Run sequencing will accelerate that process (Schmidl et al., 2015; Krishnaswami et al., 2016; Macaulay et al., 2017; Skene et al., 2018). Overall, these techniques will contribute to our understanding of the weight cycling phenomenon, will help to identify novel pathways and targets in the CNS control of body weight, and will establish whether an epigenetic basis for the Yoyo-dieting phenomenon exists.

## Author Contributions

RC, SS and PP wrote and edited the manuscript.

## Funding

This work has received funding from the European Union's Horizon 2020 Research and Innovation Programme under the Marie Skłodowska-Curie Grant Agreement No. 675610. This work was further supported in part by the DZD (SCS, PP), by the Helmholtz-Israel-Cooperation in Personalized Medicine (PP) and through the Initiative and Networking Fund of the Helmholtz Association.

## Conflict of Interest

The authors declare that the research was conducted in the absence of any commercial or financial relationships that could be construed as a potential conflict of interest.
